# Effects of acidifiers on soil greenhouse gas emissions in calcareous soils in a semi-arid area

**DOI:** 10.1038/s41598-023-32127-0

**Published:** 2023-03-29

**Authors:** Mehdi Derafshi, Behnam Asgari Lajayer, Akbar Hassani, Bernard Dell

**Affiliations:** 1 Department of Soil Science, Faculty of Agriculture, University of Zanjan, Zanjan, Iran; 2 Department of Soil Science, Faculty of Agriculture, University of Tabriz, Tabriz, Iran; 3 Agriculture Sciences, Murdoch University, Murdoch, 6150 Australia

**Keywords:** Biogeochemistry, Climate sciences, Environmental sciences

## Abstract

In most agricultural fields, when soil pH is high, elemental sulfur or sulfuric acid are used to reduce soil pH and increase the availability of macro and micronutrients for optimum crop yield. However, how these inputs impact soil greenhouse gas emissions is unknown. This study aimed to measure the amount of greenhouse gas emissions and pH after the application of various doses of elemental sulfur (ES) and sulfuric acid (SA). Using static chambers, this study quantifies soil greenhouse gas emissions (CO_2_, N_2_O, and CH_4_) for 12 months after the application of ES (200, 400, 600, 800, and 1000 kg ha^−1^) and SA (20, 40, 60, 80 and 100 kg ha^−1^) to a calcareous soil (pH 8.1) in Zanjan, Iran. Also, in order to simulate rainfed and dryland farming which are common practices in this area, this study was conducted with and without sprinkler irrigation. Application of ES slowly decreased soil pH (more than half a unit) over the year whereas application of SA temporarily reduced the pH (less than a half unit) for a few weeks. CO_2_ and N_2_O emissions and CH_4_ uptake were maximum during summer and lowest in winter. Cumulative CO_2_ fluxes ranged from 1859.2 kg^−1^ CO_2_-C ha^−1^ year^−1^ in the control treatment to 2269.6 kg CO_2_-C ha^−1^ year^−1^ in the 1000 kg ha^−1^ ES treatment. Cumulative fluxes for N_2_O-N were 2.5 and 3.7 kg N_2_O-N ha^−1^ year^−1^ and cumulative CH_4_ uptakes were 0.2 and 2.3 kg CH_4_-C ha^−1^ year^−1^ in the same treatments. Irrigation significantly increased CO_2_ and N_2_O emissions and, depending on the amount of ES applied, decreased or increased CH_4_ uptake. SA application had a negligible effect on GHGs emissions in this experiment and only the highest amount of SA altered GHGs emissions.

## Introduction

Agricultural soils are responsible for 18% of global greenhouse gas emissions^1^. This amount is predicted to increase due to the global population growth and increasing demand for food, all adding pressure to the agriculture practices to increase productivity. The concentration of greenhouse gases (GHGs), such as carbon dioxide (CO_2_), nitrous oxide (N_2_O) and methane (CH_4_), is rising rapidly^2–5^, resulting in climate change, altered precipitation regimes and global warming^6,7^. Carbon dioxide is the primary GHG, but N_2_O and CH_4_ also have a significant influence on climate change, while being released in smaller quantities than CO_2_. Methane and N_2_O have 25 and 298 times more global warming potential than CO_2_ over a 100-year time horizon, respectively^8^. The application of fertilizer is one of the main reasons for increasing GHGs emissions in crop production^9^.

Calcareous soils cover more than 30% of the earth’s land surface^10^. In calcareous soil, the soil inorganic carbon (SIC) pool includes not only calcium carbonate (CaCO_3_) and magnesium carbonate (MgCO_3_) deposited as solid phase in the soil^11^, but also HCO_3_ in soil solution (liquid phase), and CO_2_ in the soil pores (gas phase)^12^. In earlier studies, the importance of SIC in measuring CO_2_ emissions has often been neglected due to the fact that SIC is more stable than that soil organic carbon (SOC). However, with developments in isotope technology for C cycle analysis, it was found that SIC is a C source^13^. The SIC stocks are most abundant in calcareous soils of arid and semi-arid regions, where SIC is responsible for 27% of total CO_2_ emissions^14^. Consequently, SIC stability in calcareous soils has a significant effect on CO_2_ in the atmosphere and the C balance^14^.

Most of the agricultural land in Iran is characterized by calcareous soils that contain relatively high amounts of CaCO_3_ with low organic matter resulting in a pH above 7, and reduced availability of elements essential for plant growth such as phosphorus, iron and manganese^15^. Optimum soil pH is a key factor determining crop yield and quality^16^ and most agricultural plants yield optimally at a pH range of 6.5 to 7.5^17^. Sulfur is commonly used to acidify alkaline soils^18^. Ferrous sulfate, aluminum sulfate and elemental sulfur can be used to lower soil pH. Elemental sulfur (ES) applications have been shown by many studies to have long-lasting effects and be economically practicable for soils high in CaCO_3_^19–21^. Elemental sulfur application is a viable option for neutralizing soil pH in many areas^22^ and it has been shown to provide balanced fertilization for crops and improve crop yield if applied in appropriate amounts^23^. Since there are many sulfur mines in Iran and sulfur is one of the by-products of gas refineries, due to its cost-effectiveness, most farmers use elemental sulfur as an acidifier to reduce soil pH prior to cropping.

Farmers typically use between 200 and 500 kg of ES per hectare. The ES is most likely converted to H_2_SO_4_ in the soil by acidophilic microorganisms such as *Acidithiobacillus thiooxidans* according to Eq. (1). Sulfuric acid reacts with CaCO_3_ in the soil releasing CO_2_ according to Eq. (2). In this way, farmers help release CO_2_ into the atmosphere.

1 $${\text{S}} + {\text{O}}_{{2}} \to {\text{ SO}}_{{2}} + {\text{ O}}_{{2}} + {\text{ H}}_{{2}} {\text{O }} \to {\text{ SO}}_{{3}} + {\text{ H}}_{{2}} {\text{O }} \to {\text{ H}}_{{2}} {\text{SO}}_{{4}} ,$$

2 $${\text{H}}_{{2}} {\text{SO}}_{{4}} + {\text{ CaCO}}_{{3}} \to {\text{ CaSO}}_{{4}} + {\text{ H}}_{{2}} {\text{O }} + {\text{ CO}}_{{2}} .$$

Up to now, GHGs emissions from calcareous soils has not been fully investigated and data regarding the effect of land management and environmental condition (soil properties such as moisture, pH) on calcareous soil GHGs emissions are scarce. Only a few studies have investigated the effects of biochar and compost on GHGs emissions in calcareous soil^24–26^ and there were no other studies on GHG emissions from calcareous soils where the pH has been modified. Therefore, this study aimed to investigate the extent to which the addition of elemental sulfur or sulfuric acid to a calcareous soil alters the emissions of CO_2_, NO_2_ and CH_4_. The emission of GHGs was quantified over 12 months in the field.

## Materials and methods

### Site description

The experiment was conducted from March 2021 to February 2022 in the Research Farm of the University of Zanjan, Zanjan, Iran at 1663 m elevation. This semi-arid area has an average of 250 mm of precipitation, mainly from November to May, and the average annual temperature is 13 °C, ranging from − 8 in January and February to 31 °C in July and August. In the past 3 years wheat was grown on this land. The land was kept fallow during the course of the trial. A one-hectare field was chosen with soil properties typical of productive agricultural land in Iran. The soil had a pH before treatment of 8.14. Other soil properties are given in Table S1.

### Trial design and treatments

**Figure 1:**
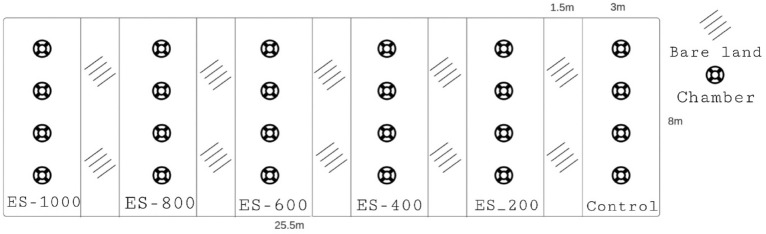
Layout of greenhouse gas flux measurement rings for each trial area.

A field experiment was set up comprising 5 rates of ES, five rates of H_2_SO_4_ and an untreated control, the rates were chosen from local experience of agricultural practices (Table 1). Four similar trial areas (204 m^2^) were chosen, one for ES without irrigation, one for ES with irrigation, one for SA without irrigation, and one for SA with irrigation. Each trial area comprised 6 plots (each plot was 24 m^2^) without replication (Fig. 1). Elemental sulfur with 99% purity with a particle size of 75 microns was purchased from Bandar Abbas Refinery. The powder was distributed on the soil surface and integrated into the soil with a rotating cultivator to a depth of 20 cm on 22th of February, and immediately, PVC rings were placed on the field for GHGs measurement. Sulfuric acid with 98% purity purchased from the local market. The required amounts of sulfuric acid were diluted in 1 cubic meter of fresh water and then applied to the field by sprinkler irrigation between 8 and 9 AM on March the first. Unlike for ES, the soil was not cultivated after treatment.

**Table 1:** Treatment details.

Treatment	Code	Rate per hectare (kg)
Control		
Elemental sulfur	ES-200	200
Elemental sulfur	ES-400	400
Elemental sulfur	ES-600	600
Elemental sulfur	ES-800	800
Elemental sulfur	ES-1000	1000
Sulfuric acid	SA-20	20
Sulfuric acid	SA-40	40
Sulfuric acid	SA-60	60
Sulfuric acid	SA-80	80
Sulfuric acid	SA-100	100

As there is dryland and irrigated farming in this region of Iran, two water regimes were used: no irrigation for simulating rainfed cultivation and sprinkler irrigation for simulating irrigated farming. Sprinkler irrigation was applied at 7-day intervals from May 5th to 13th October. At each application, 5 m^3^ of fresh water was supplied to a 204 m^2^ field.

### Greenhouse gas flux measurements

First, 96 PVC rings (base: diameter: 27 cm, height: 10 cm) were inserted with a rubber hammer 8 to 10 cm into the soil (the chambers were 2 m apart) and remained in place throughout the experiment. Rings were placed in the soil after the application of ES on February 22nd or before the application of SA. Then, soil temperature and moisture sensors (KIMO HQ-210, Kimo Instruments, France) were inserted at a distance of 3–6 cm around the rings. Homemade Closed static chambers were placed onto the PVC rings (lid: diameter: 27 cm, height: 37 cm) and sealed with a white polyurethane spray foam. Each chamber top was equipped with a Luer-lock interface and a valve.

Gas samples were taken every 7 days in the morning between 08:00 and 11:00 h. Gas samples were taken 4 times over a total of 30 min (0, 10, 20 and 30 min) using 20 mL Luer-lock syringes with ≤ 0.3 mm needles. Initially, syringes were filled and re-injected thrice, in order to prepare syringes for samples. Then, the chamber and syringe valves were closed and syringes were detached. Next, gas samples were injected with a needle into the glass vials and stored in a secure box with a vial separator and transported to the laboratory for gas analysis. The gas samples were analyzed 2 to 5 days after field sampling.

SRI 8610c gas chromatography (SRI Instruments, Torrance, CA, USA) was used to quantify CO_2_, N_2_O, and CH_4_ concentrations in the samples. The GC was equipped with a Flame Ionization Detector (FID-Methanizer) for CO_2_ and CH_4_ detection and a ^63^Ni Electron Capture Detector (ECD) to measure N_2_O. Nitrogen (20 mL N_2_ min^−1^) was used to reduce the detector noise. Before measuring samples, and every 6 samples, the GC was calibrated with a standard mixture of pure CH_4_, N_2_O, and CO_2_, and a calibration curve was built. Peak areas were used to obtain CO_2_, N_2_O and CH_4_ concentrations in every sample. Linear regression was used to calculate CO_2_ and CH_4_ concentrations and a power function for N_2_O fluxes. Chamber fluxes were converted to µg gas m^−2^ h^−1^ using the ideal gas law (n = PV/RT) where P was pressure equal to 1 atm, V (0.02 m^3^) was the volume of the chamber, R was 0.082057 l atm/(K^.^mole), and T was the temperature in the field (K) and moles were converted to g by multiplying molecular weight of the gases.

### Soil pH monitoring

In order to measure pH, soil (5–10 cm) from 10 different places in each plot were gathered every 7 days, combined together and a sub-sample used in a 1:2.5 soil: water ratio to determine the pH with a Jenway 3510 pH meter.

### Statistical analysis

Statistically differences in CO_2_, N_2_O, CH_4_ and pH between treatments were determined using two-way repeated measures ANOVA at 0.05 probability followed by Duncan’s multiple range test using SPSS Version 22. Also, Pearson correlation was used to identify any correlations between GHGs emissions and variables including soil moisture, soil temperatures and air temperature. In order to calculate cumulative gas emissions, the average of two consecutive determinations were multiplied by the time interval between them and then added to the prior cumulative total^27^. The difference between cumulative GHGs emissions among the six treatments was analyzed by one-way ANOVA and LSD test with 0.05 probability.

## Results and discussion

### Effects of acidifiers on soil pH

**Figure 2:**
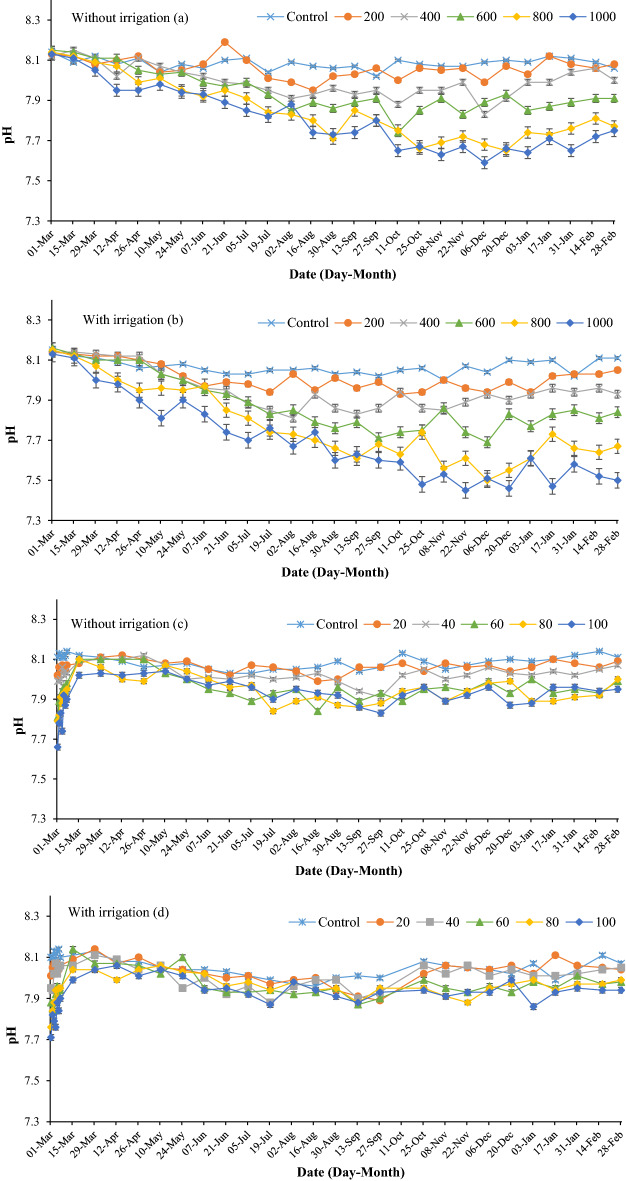
Effects of elemental sulfur and sulfuric acid on soil pH in non-irrigated ((**a,c**), respectively) and irrigated fields ((**b,d**), respectively). The values were expressed as mean ± standard deviation (SD).

The higher rates (800 and 1000 kg ha^−1^) of ES lowered soil pH over time in spring and summer (Fig. 2a). The soil pH decreased from 8.13 in March to 7.65 in October and then stabilized. The three lower ES treatments (200, 400 and 600 kg) did not significantly lower soil pH value in the non-irrigated treatments (Fig. 2a). The pH declined more significantly in the irrigated treatments in response to ES application. The lowest pH was recorded in the 1000 kg ES, where the pH reached 7.5 after a year (Fig. 2b). It is clear that irrigation can increase the effectiveness of ES application. Notably, after commencing irrigation on 5th May, the pH began to fall more rapidly than in the non-irrigated treatments during the same period.

The increase in soil moisture following irrigation can stimulate the activity of soil microorganisms including *Thiobacillus* spp. resulting in greater conversion of ES to sulfuric acid^28^. Jaggi et al.^29^ concluded that with increasing soil moisture, the efficiency of ES on lowering soil pH is improved. Moreover, water can expedite the distribution of ES into the soil matrix, thus improving the accessibility of microbes to ES^30^.

Elemental sulfur oxidization is a slow process, and depends on many factors such as temperature, soil moisture, sulfur particle size, pH, activity of soil microbes, and soil fertility^31^. Sameni and Kasraian^32^ found that elemental sulfur took a long time to decrease soil pH and concluded that this was due to slow biological processes in the soil. In contrast, Abou Hussien et al.^33^ applied 200 and 400 kg ES to soil and achieved a more significant decrease in soil pH than in the present study. This can be explained by the soil in their study having more sand (78.5%), lower CaCO_3_ content (13.9%), and less organic matter (0.55%) than in this trial. Janzen and Bettany^34^ found a strong, positive and significant correlation between the rate of sulfur oxidation and the amount of organic matter in the soil.

The steeper decrease in soil pH in summer could be a consequence of higher temperatures increasing the activity of soil microbes^35^. Also, the rate of sulfur oxidation increases with ambient temperature^36^.

The effect of addition of SA on soil pH was faster than ES, as the soil pH fell rapidly shortly after being applied (Fig. 2c,d). In the SA-100 treatment the soil pH declined to 7.66, but after 2 weeks the pH returned to the starting pH of 8.02. However, from June (summer) the soil pH was slightly reduced compared to the control. Unlike for ES application where irrigation caused greater decline in pH compared to non-irrigated treatments, there was no significant difference in pH between the control and SA-100 treatment under irrigation. Overall, SA application effects on soil pH were not long-lasting and, in order to lower soil pH for the entire season, SA would need to be applied regularly. Furthermore, ES should be applied a few months before seeding to reach the desired pH since ES oxidization is a time-consuming process.

### CO_2_ emissions

**Figure 3:**
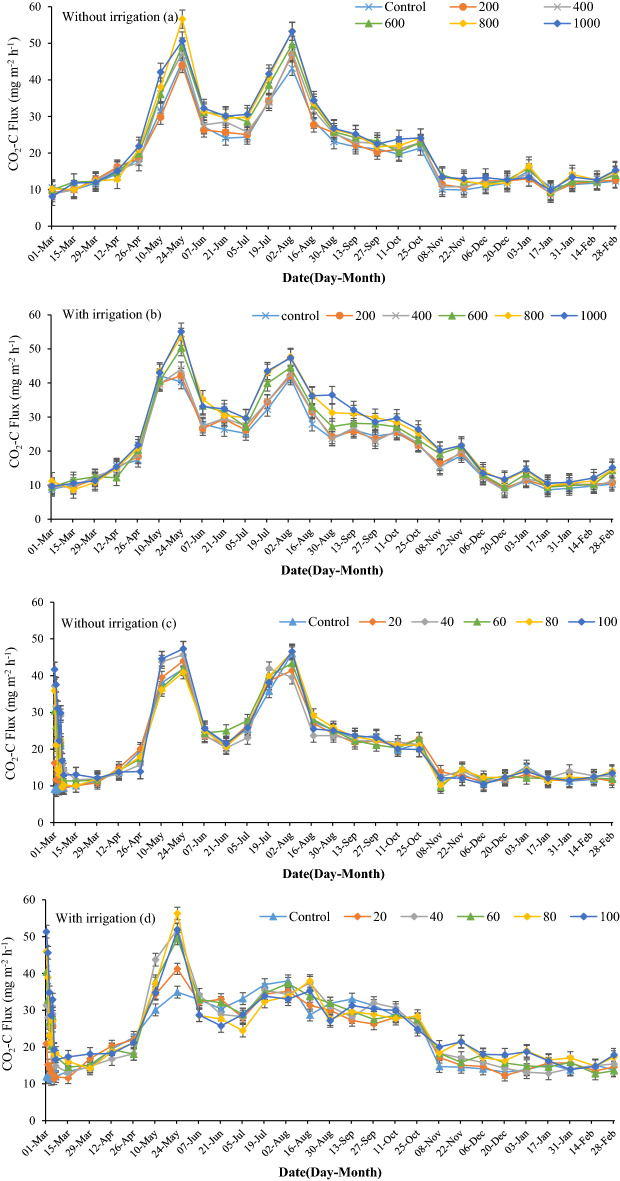
Effects of elemental sulfur and sulfuric acid on CO_2_ emissions in non-irrigated ((**a,c**), respectively) and irrigated fields ((**b,d**), respectively). The values were expressed as mean ± standard deviation (SD).

There was no significant difference in CO_2_ emission between ES treatments early in the experiment and in winter, but in spring and summer, CO_2_ fluxes were higher in ES applied treatments compared to the control (Fig. 3a,b). For example, during the spring–summer period the CO_2_ fluxes in the ES-1000 treatment were 4 to 10 CO_2_-C mg m^−2^ h^−1^ greater than the control treatment. The highest CO_2_ flux (55 mg CO_2_-C m^−2^ h^−1^) was on 24th May after rainfall (17 mm) following a long dry period. Similarly, there was another peak in August following rainfall (8 mm). The average CO_2_ fluxes were between 27 and 32 mg CO_2_-C m^−2^ h^−1^ in summer in non-irrigated fields, but in winter the average CO_2_ fluxes ranged from 11.7 to 12.9 mg CO_2_-C m^−2^ h^−1^. However, there was no significant difference in mean CO_2_ fluxes between treatments over the year.

Xiao et al.^37^ recorded a similar trend in CO_2_ emissions in a calcareous soil. In their study, around 30 mg m^−2^ h^−1^ CO_2_ occurred in winter (November to February) and around 40.9 mg CO_2_-C m^−2^ h^−1^ emissions occurred in summer (May to September). Also, the average CO_2_ emissions in the present study (20.4 mg CO_2_-C m^−2^ h^−1^) is similar to their study (21.9 mg CO_2_-C m^−2^ h^−1^). Furthermore, low rates of ES had no effect on GHGs emissions^38^, which is similar to our study.

The trends of CO_2_ fluxes in the irrigated field were similar to the non-irrigated field, but CO_2_ fluxes varied significantly between treatments (Fig. 3a). Also, there was a notable difference in CO_2_ fluxes between the irrigated and non-irrigated plots during the irrigation period (5th May to 13th October). The average CO_2_ flux increased by around 2.7 mg CO_2_-C m^−2^ h^−1^ year^−1^ as a result of irrigation. However, after the irrigation period, from November to February, the difference in CO_2_ fluxes between irrigated and non-irrigated plots diminished. The mean CO_2_ flux in the control treatment was 20.4 mg CO_2_-C m^−2^ h^−1^ year^−1^ and 24.9 in the ES-1000 treatment.

Sulfuric acid increased CO_2_ fluxes immediately after application and one of the highest peaks was observed on the first day of application (Fig. 3c,d). However, the CO_2_ flux declined and reached the normal flux rate after 2 weeks. In non-irrigated treatments, peaks occurred after the rain events as in the ES results, but there was only one peak in the irrigated plots (Fig. 3d). Overall, SA application had a small effect on the average CO_2_ flux and there was only a 0.5 mg CO_2_-C m^−2^ h^−1^ flux difference between the control and SA-100 treatments. Hence, there was no significant difference in mean CO_2_ emissions between treatments.

**Figure 4:**
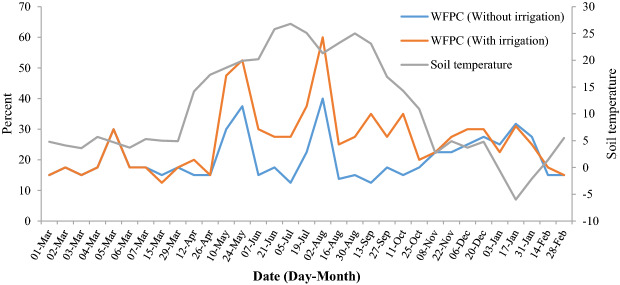
Soil temperature and water filled pore space (WFPC) of the experimental field.

Factors driving peaks in soil CO_2_ emission include soil moisture, soil temperature and fertilizer use^39–41^. Soil moisture is the most important factor regulating soil gas fluxes^42,43^, and positive correlations between soil moisture and CO_2_ emissions are well documented^37,44,45^. CO_2_ emission decreases during drought, but after precipitation a pulse in CO_2_ emission occurs, known as the Birch Effect^11^. However, with more frequent wet-dry cycles, the Birch effect diminishes^46^, and this might be the reason for a lower peak (on August second) in irrigated treatments. In other words, between the two peaks in irrigated treatments, the soil WFPS was around 30%, but in non-irrigated treatments, it was approximately 15% (Fig. 4). CO_2_ emissions increase for short periods (minutes to days) before returning to the usual level^47^. In the present study, the mean difference in soil water-filled pore space in the irrigation period between irrigated and non-irrigated treatments was 15% (Fig. 4). Moreover, Zhang et al.^44^ showed that CO_2_ fluxes were significantly higher during hot-wet than during cold-dry periods. Under hot-wet conditions, more SOC is decomposed by microbes, and more CO_2_ is released into the atmosphere^48^.

In addition, the soil pH influences CO_2_ emissions. It has been observed that CO_2_ emissions were maximum in soil of neutral pH^49^. The large gap between the control treatment and the ES treatment in summer can be related to lower soil pH, higher sulfur oxidization, and higher microbial population and activity. Also, when irrigation occurred, this gap widened as a result of more microbial activity and higher soil moisture. After application of ES the population of *Thiobacillus* spp. and aerobic heterotrophic S-oxidizing bacteria increased^50,51^.

### N_2_O emissions

**Figure 5:**
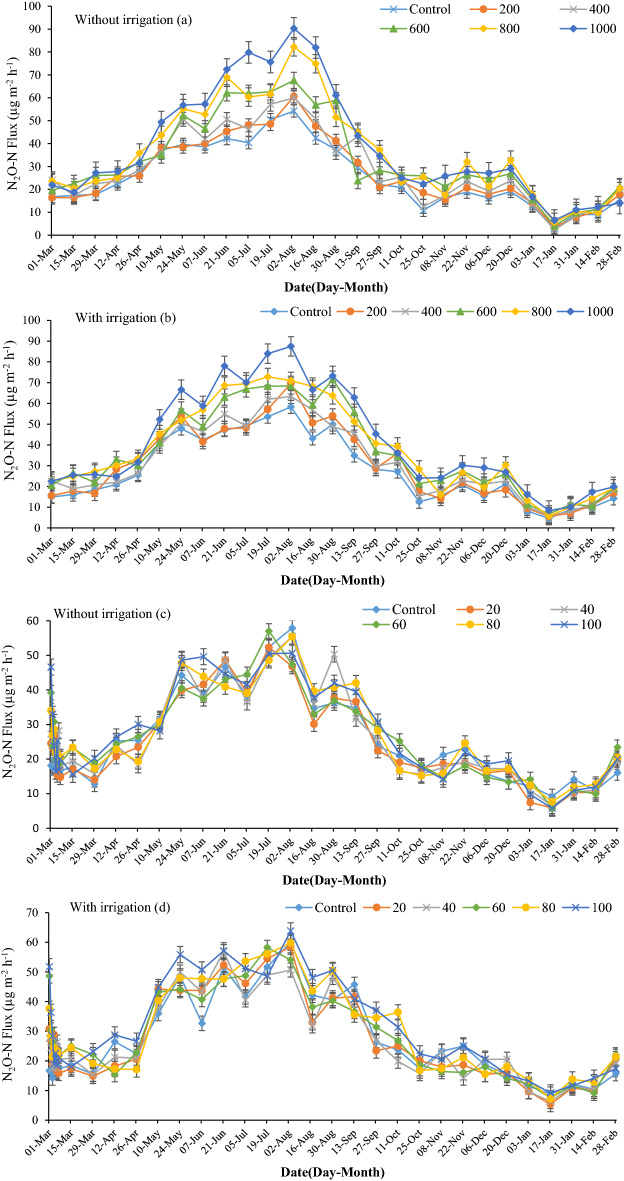
Effects of elemental sulfur and sulfuric acid on N_2_O emissions in non-irrigated ((**a,c**), respectively) and irrigated fields ((**b,d**), respectively). The values were expressed as mean ± standard deviation (SD).

Nitrous oxide fluxes were positive throughout the year for all treatments (Fig. 5). The fluxes were highest in summer and lowest in winter, similar to other studies^52^. The application of ES and SA increased the release of N_2_O. In the ES treatments, irrigation caused N_2_O emissions to increase compared to non-irrigated fields. The mean annual N_2_O emissions were 25.4 N_2_O-N µg m^−2^ h^−1^ in the control treatment and 38.7 in the ES-1000 treatment. Notably, this gap widened with irrigation, which were 27.3 and 41.3 µg N_2_O-N m^−2^ h^−1^ in the control and ES-1000 treatments, respectively. As the soil texture was sandy, even a small rain event had a significant effect on N_2_O emission, reaching the highest amount (90.3 µg N_2_O-N m^−2^ h^−1^) in early August in both irrigated and non-irrigated fields. However, precipitation in winter did not significantly affect N_2_O fluxes, as precipitation occurred as snow and remained on the soil surface for days due to low temperatures. The N_2_O fluxes in the irrigation period (May 5th to October 13th) differed significantly between irrigated and non-irrigated treatments. The average N_2_O fluxes were 25.3, 27.1, 29.2, 33.1, 36.2, and 38.7 µg N_2_O-N m^−2^ h^−1^ in the control, ES-200, ES-400, ES-600, ES-800, and ES-1000 treatments in non-irrigated plots, respectively, but this increased to 27.7, 30.4, 31.1, 36.4, 37.8, and 41.3 in the same treatments in the irrigated plots, respectively (Fig. 5a,b).

Application of sulfuric acid had no effect on N_2_O fluxes in most treatments (Fig. 5c,d). Only the SA-100 treatment significantly increased mean annual N_2_O fluxes compared to the control treatments in both irrigation regimes. The annual trend for N_2_O fluxes was similar to the ES treatments, gradually increasing in spring and reaching a peak in summer, followed by a decline in fall and finally the lowest emission in winter.

Irrigation increased N_2_O fluxes compared to non-irrigated treatments. This shows that more N_2_O was released under high soil moisture. Bateman et al.^53^ Showed that at 35–60% WFPS, nitrification is the main process of producing N_2_O-N. Also, ES in soil can improve the denitrification process, as ES can be used as an electron donor^54^.

In addition to soil water and ES effects, high soil and air temperatures can enhance N_2_O emission, as a result of higher soil respiration and microbial activity. Also, increasing soil temperature can reduce the O_2_ concentration in soil, resulting in higher N_2_O emissions^55^. Another reason for the difference in N_2_O emissions between the control treatment and the ES treatments is that ES can stimulate denitrification and dissimilatory nitrate reduction to ammonium, performed by species of *Thiobacillus* and *Nocardioides*^56,57^.

### CH_4_ emissions

**Figure 6:**
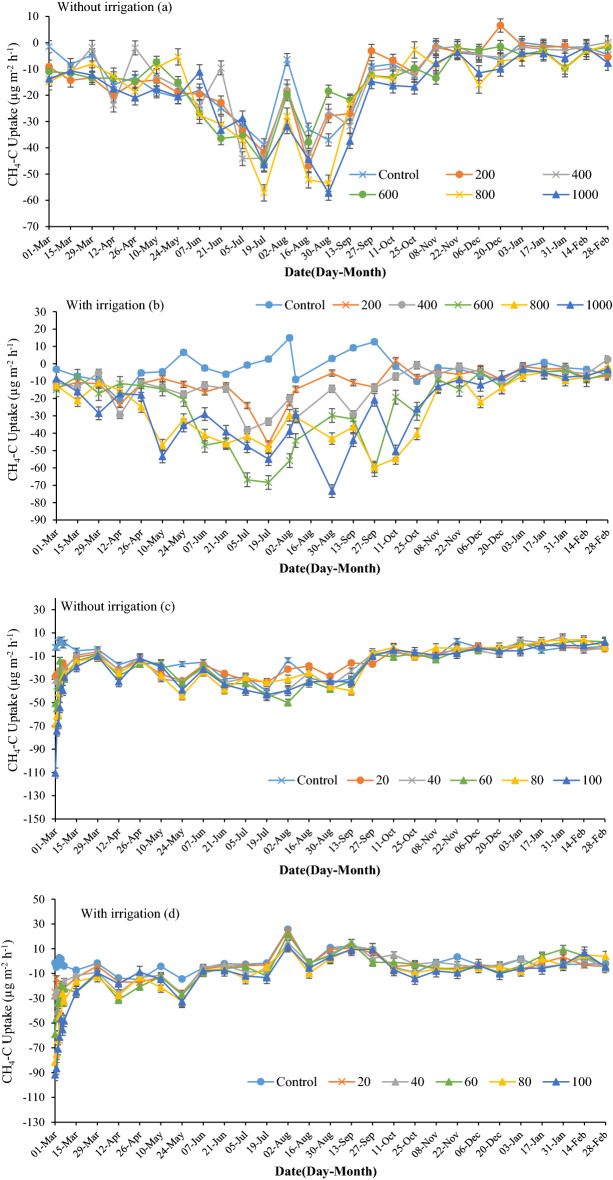
Effects of elemental sulfur and sulfuric acid on CH_4_ emissions in non-irrigated ((**a,c**), respectively) and irrigated fields ((**b,d**), respectively). The values were expressed as mean ± standard deviation (SD).

In most treatments, soil acted as a CH_4_ sink throughout the year (Fig. 6). In the non-irrigated field, CH_4_ uptake increased gradually in spring, to a maximum on 30th August of 57.1 µg CH_4_-C m^−2^ h^−1^ in the ES-100 treatment, then declined in the fall to low levels over winter (Fig. 6a). The results showed that application of ES significantly increased CH_4_ uptake in calcareous soils compared to the control treatment.

Irrigation converted the control treatment from a soil sink to an emission source of CH_4_ over the period May to September. Emissions reached 14.8 µg CH_4_-C m^−2^ h^−1^ when high temperature was accompanied by rain. However, irrigation increased the soil CH_4_ uptake rate in ES-applied treatments. It is noteworthy that before and after irrigation, there was no difference in CH_4_ emissions between irrigated and non-irrigated treatments. Some authors have reported that soil can convert from a net sink to source depending on soil properties^58–60^. For example, Jäckel et al.^61^ reported that when soil moisture decreased to less than 20% water holding capacity, soils turned from being sinks into a net source of atmospheric CH_4_.

Application of SA increased CH_4_ uptake (Fig. 6c and 6d). Uptake increased to the highest amount (110.4 µg CH_4_-C m^−2^ h^−1^ in SA-100 treatment) immediately after SA application and gradually returned to the normal level within two weeks. In the non-irrigated field, SA increased CH_4_ uptake in summer but not at other times of the year (Fig. 6c). Irrigation caused a reduction in CH_4_ uptake in all SA treatments and CH_4_ uptake increased by roughly 20 µg CH_4_-C m^−2^ h^−1^ in each treatment compared to non-irrigated treatments in the irrigation period, but there was no significant difference in reduction between treatments (Fig. 6d). Even the control treatment in the irrigation plots became a CH_4_ source with an average of 0.75 µg CH_4_-C m^−2^ h^−1^. Overall, irrigation reduced the amount of CH_4_ uptake in this study compared to non-irrigated soil.

CH_4_ is generated through methanogenesis by methanogenic bacteria in soil, which is strictly an anaerobic process^62^. Considerable CH_4_ emissions occur in wetlands and paddy fields, as well as in soils with high SOC content^63,64^. However, sulfate-rich compounds can inhibit methanogenesis in anaerobic environments, due to competition for hydrogen and acetate by sulfate-reducing bacteria^65^. Also, when sulfate reducing bacteria reduce the hydrogen concentration and CH_4_ rises above the hydrogen concentration, CH_4_ is oxidized to CO_2_ by reverse methanogenesis by archaea^66–68^.

It has been shown that high soil pH is associated with high uptake of CH_4_ in calcareous soils^69,70^. One reason could be the low soil moisture content in calcareous soils in arid and semi-arid regions, resulting in higher content of oxygen in the soil and increasing the rate of methane oxidization. Furthermore, under low oxygen content the CH_4_ can diffuse further down the soil profile^71^.

Studies have indicated that different sulfur compounds such as sulfate, sulfide, and sulfite can inhibit CH_4_ production in anaerobic conditions^72,73^. Purdy et al.^74^ showed that when soil SO_4_^2^ concentration is low, CH_4_ production is stimulated by outcompeting sulfate-reducing bacteria. Also, in the process of anaerobic oxidation of methane, sulfate is oxidized with methane as a terminal electron acceptor^75^.

### Cumulative greenhouse gas emissions

#### CO_2_

**Figure 7:**
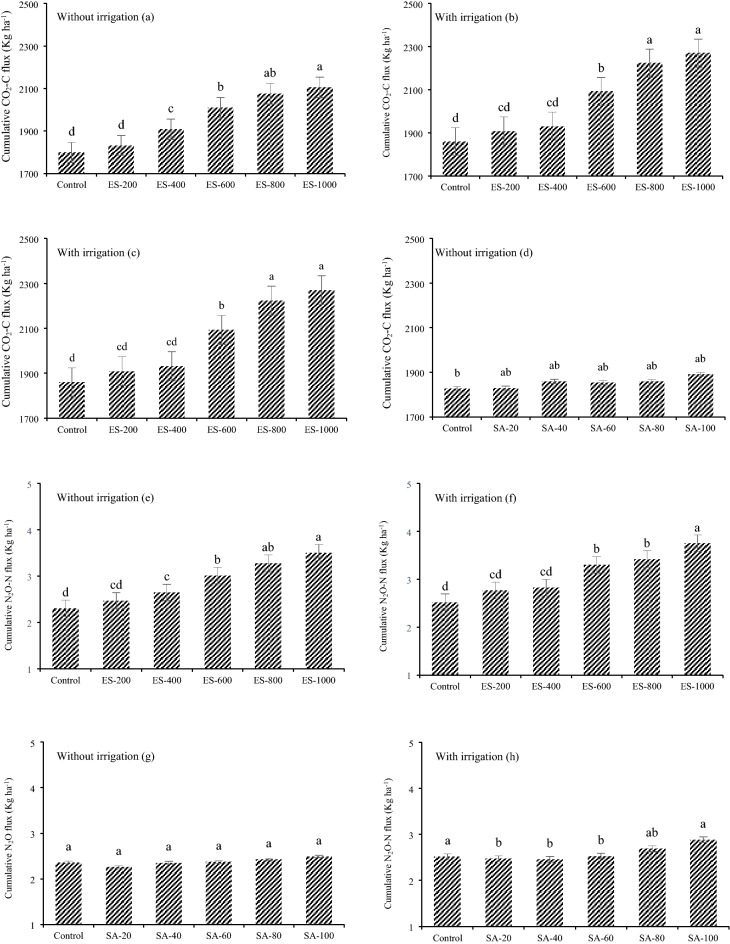

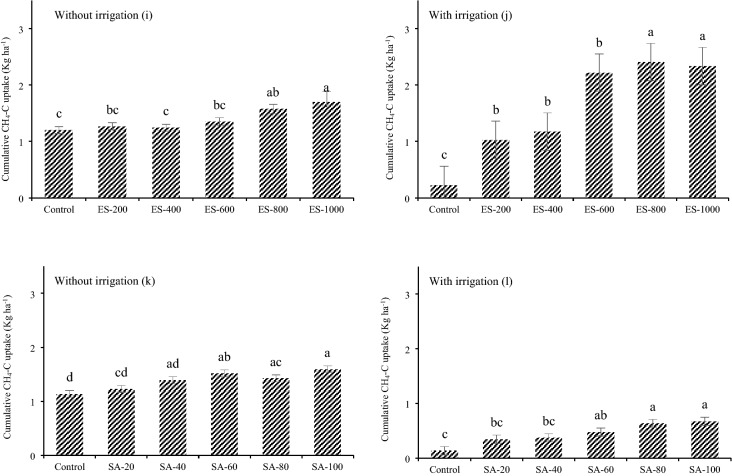
Cumulative CO_2_, N_2_O and CH_4_ emissions in non-irrigated and irrigated fields. The values were expressed as mean ± standard deviation (SD). The different letters indicate significant differences at *p* < 0.05.

The application of ES significantly increased the cumulative emissions of CO_2_ in both irrigated and non-irrigated fields. In the non-irrigated field, ES increased the cumulative CO_2_ emission from 1798.2 kg CO_2_-C ha^−1^ year^−1^ in the control treatment to 2105.5 in the ES-1000 treatment (Fig. 7). Notably, this gap widened in the irrigated field, and reached 2269.6 kg CO_2_-C ha^−1^ year^−1^ in the ES-1000 treatment. Also, cumulative CO_2_ emissions were higher in all of the irrigated treatments compared to the same treatments in the non-irrigated field.

However, application of SA did not significantly increase cumulative CO_2_ emissions in the non-irrigated field. By contrast, SA treatments from 40 to 100 increased cumulative CO_2_ emissions by 4%. Thus, irrigation as well as ES were critical driving factors for CO_2_ emissions (Fig. 7).

The CO_2_ emissions were similar to values obtained by Xiao et al.^37^ for another calcareous soil where the cumulative CO_2_ flux was 1914.5 kg CO_2_-C ha^−1^ year^−1^.

#### N_2_O

Similar to the CO_2_ emission, cumulative N_2_O emission increased in response to the ES application. Also, with irrigation, the extent of N_2_O emission increased compared to the non-irrigated treatments. However, the difference between the control treatment and ES-1000 treatment was 1.19 kg N_2_O-N ha^−1^ but with irrigation this difference in the same treatments was 1.2 kg N_2_O-N ha^−1^, which shows the extent of ES oxidization did not significantly affect N_2_O emission and only the irrigation affected N_2_O emission. In other words, as soil moisture increases the conversion of ES to SA, this conversion has no effect on N_2_O emission and the difference between N_2_O emission between irrigated and not irrigated plots can be explained by high soil moisture.

Nevertheless, SA application did not affect N_2_O emission in the non-irrigated field and only one treatment (SA-100) with irrigation increased it significantly to 2.86 kg N_2_O-N ha^−1^ compared to the control treatment of 2.4 kg N_2_O-N ha^−1^. However, all the treatments in the irrigated field had higher cumulative N_2_O emissions compared to the non-irrigated treatments (Fig. 7). Therefore, it can be concluded that soil moisture affected N_2_O emissions and in higher soil moisture more N_2_O is released to the atmosphere.

#### CH_4_

Application of ES increased cumulative CH_4_ uptake in the non-irrigated field. In the control treatment, 1.59 kg CH_4_ ha^−1^ year^−1^ was absorbed from the atmosphere and this amount increased to 1.1 kg CH_4_-C ha^−1^ year^−1^ in the ES-1000 treatment. However, in the irrigated field less CH_4_ was absorbed from the atmosphere (0.2 kg CH_4_-C ha^−1^ year^−1^). Interestingly, a considerable difference between ES-400 and ES-600 was witnessed with 1.1 and 2.1 kg CH_4_-C ha^−1^, respectively, increasing to 2.3 kg CH_4_-C ha^−1^ in the ES-1000 treatment. Furthermore, cumulative CH_4_ uptake in the first 3 treatments (control, ES-200, ES-400) in the irrigated field was less than the uptake by the non-irrigated soil, but CH_4_ uptake in the 3 remaining treatments (ES-600, ES-800, ES-1000) in the irrigated field were greater than in the non-irrigated field.

Similar to ES application, SA increased CH_4_ uptake in the non-irrigated field. This difference in lower amounts of SA was insignificant, but as the amount of SA increased, a greater amount of CH_4_ was absorbed. Irrigation significantly reduced the CH_4_ uptake in the irrigated field compared to the non-irrigated field. Compared to the control treatments (with and without irrigation), the reduction was more than 8 times, from 1.1 to 0.07 kg CH_4_-C ha^−1^. Also, in SA applied treatments, CH_4_ uptake was reduced at least 2 times in the irrigated compared to non-irrigated field (Fig. 7).

Soil microbes are responsible for release and uptake of CH_4_ in soil; CH_4_ fluxes are the outcome of aerobic soil microbe activity, whereas CH_4_ uptake is the outcome of anaerobic microbe activity^76^. Comparing the control treatments in the irrigated and non-irrigated treatments, high soil moisture as a result of irrigation decreased CH_4_ uptake. However, when ES was added to the soil, this pattern changed. At the lower ES rates (200 and 400 kg ha^−1^), irrigation reduced CH_4_ uptake, but at higher rates (600, 800, and 1000 kg ha^−1^) irrigation increased CH_4_ uptake. The reason behind this might be that when soil sulfur is low, high soil moisture can help release CH_4_. However, when soil sulfur content increases, the anaerobic environment cannot improve CH_4_ fluxes due to the presence of sulfur containing compounds.

### Effects of pH, soil moisture, soil temperature, and air temperature on CO_2_, N_2_O and CH_4_ emissions

In ES applied treatments, GHGs emissions were not positively correlated with soil pH (Table 2). Similarly, Hu et al.^77^ showed pH can potentially affect CO_2_ emissions, but no correlation was found between CO_2_ emissions and pH.

**Table 2:** Correlation between GHGs emissions and soil pH, moisture, temperature and air temperature.

Irrigation regime	Treatment		CO_2_	N_2_O	CH_4_
Without irrigation	Control	Soil pH	− 0.37	− 0.34	0.38*
	ES-200		− 0.37	− 0.25	0.23
	ES-400		− 0.20	− 0.19	0.21
	ES-600		− 0.05	0.02	− 0.09
	ES-800		0.09	0.13	− 0.03
	ES-1000		0.2	0.2	− 0.10
With irrigation	Control		− 0.41*	− 0.41*	− 0.36
	ES-200		− 0.27	− 0.19	− 0.04
	ES-400		− 0.32	− 0.32	0.1
	ES-600		− 0.07	− 0.03	0.18
	ES-800		0.04	0.11	− 0.04
	ES-1000		0.06	0.15	− 0.14
Without irrigation	Control		− 0.60**	− 0.72**	0.70**
	SA-20		− 0.18	− 0.37*	0.30
	SA-40		− 0.36	− 0.37*	0.29
	SA-60		0.28	− 0.23	0.34
	SA-80		− 0.17	− 0.12	0.33
	SA-100		− 0.22	− 0.05	0.62
With irrigation	Control		− 0.62**	− 0.62**	− 0.10
	SA-20		− 0.51**	− 0.55**	0.33
	SA-40		− 0.55	− 0.63**	0.37*
	SA-60		− 0.2	− 0.20	0.40*
	SA-80		− 0.23	− 0.03	0.73**
	SA-100		− 0.35*	0.01	0.78**
Without irrigation	Soil moisture		0.38	0.15	0.24
	Soil temperature		0.8**	0.80**	− 0.82*
With irrigation	Soil moisture		0.82**	0.67**	0.60
	Soil temperature		0.84**	0.81**	0.46*
	Air temperature		0.76**	0.89**	− 0.87**

*p < 0.05, **p < 0.01.

Only CH_4_ emissions in non-irrigated and N_2_O and CO_2_ in irrigated treatments were positively correlated with pH with the control treatment (p < 0.05). However, with SA more treatments were positively correlated with soil pH. Not only GHGs emissions in control treatments were positively correlated with pH, but also the soil SA applied treatments were positively correlated with pH (p < 0.01). It seems pH has is more correlated with N_2_O and CH_4_ emissions than CO_2_ emissions.

Moreover, there was no correlation in GHGs emission and soil moisture in non-irrigated treatments, but in irrigated treatments CO_2_ and N_2_O emissions were positively correlated with soil moisture (p < 0.01) (Table 2). What is more, all GHGs emissions were positively correlated with soil temperature (p < 0.01) in both irrigation regimes. Also, GHGs emission were positively correlated with air temperature (p < 0.01).

Soil moisture and temperature are critical driving factors of microbial biomass carbon resulting in a variation of GHGs emission among different irrigation regimes and seasons^78^. The present study also showed that GHGs emission are considerably correlated with these two factors, and similar to Ref.^37^, the effect of soil temperature was greater than soil moisture on GHGs emissions. Also, soil temperature can affect soil microorganisms and enzyme activities together with soil moisture^79,80^.

Some studies have observed a positive correlation between GHGs emissions and soil moisture and temperature in calcareous soils in semi-arid areas^37,52,81^.

Some researchers have pointed out that soil can act as a CH_4_ sink in semi-arid areas for mitigating global warming^63^. Although in the present study soils were CH_4_ sinks, the amount of CO_2_ released as a consequence of the application of acidifiers compensated for the CH_4_ uptake. In other words, the difference in CH_4_ uptake between the control and ES-1000 treatments in the irrigated field was 2.1 kg CH_4_-C year^−1^ ha^−1^, and the application of ES helped the absorbance of CH_4_ into the soil from the atmosphere. However, the difference in CO_2_ emissions in the same treatments was 410.1 kg CO_2_-C year^−1^ ha^−1^. Therefore, we cannot hypothesize that the application of acidifiers on calcareous soils in semi-arid areas can increase CH_4_ uptake substantially and mitigate the global warming rate.

### pH variation

Soil pH at the maximum rate of ES declined from 8.1 to 7.5 after 6 months of application. Many crops yield optimally at around a pH of 7.5 and lower. However, in this study, a pH of 7.5 was only reached at the end of the cultivation season. Therefore, ES should be applied a reasonable amount of time before crop establishment to reach the desired pH value. Also, it is recommended to apply ES every 3 or 4 years to prevent any pH increase. Studies have shown that elemental sulfur can improve the photosynthetic and transpiration rates of wheat and subsequently increase the straw and grain yield of wheat^82,83^. Also, wheat can yield optimally at a pH of around 6.3^84^. Moreover, SA’s effect on soil pH was not permanent and it has been shown to have dire consequences on soil health^85^. However, if SA is applied with every irrigation and in low doses, a desired level of pH can be achievable without damaging soil health. Our study was undertaken in fallow fields and so we were not able to determine the extent if any to which the root system might contribute to the amelioration of soil alkalinity. This should be taken into account in further field studies.

### Selection of soil acidifier

In order to choose the perfect strategy for lowering soil pH some factors should be considered. The application of ES is fairly easy and less equipment is required and is cost-effective compared to SA application and its effects on soil pH are permanent. However, it emits more GHGs into the atmosphere compared to SA. On the other hand, SA application does not significantly change GHGs emissions, but pH does not lower permanently and if applied regularly can be costly and more equipment is needed. Overall, in this region farmers prefer to use ES due to the above-mentioned advantages.

## Conclusions

In this study, we focused on the effects of elemental sulfur and sulfuric acid on greenhouse gas emissions on calcareous soil of a semi-arid region in Zanjan, Iran. We found that elemental sulfur application significantly lowered soil pH over several months and irrigation helped further reduce the pH. It should be considered that to reach the optimal pH before cropping, ES should be applied 6 months prior to seeding. By contrast, application of SA did not significantly reduce soil pH in the long-term. It is suggested that for reducing soil pH with SA, frequent SA application is necessary. CO_2,_ N_2_O and CH_4_ emissions were significantly affected by elemental sulfur application especially in irrigated treatments, where CO_2_ and N_2_O fluxes increased and CH_4_ uptake increased. However, only the highest rate of sulfuric acid application (SA-100) considerably affected N_2_O and CH_4_ fluxes and no significant difference in CO_2_ emissions were observed. The results of this study will help scientists to have better understanding of the effects of acidification, irrigation and fertilizer application on GHGs emissions in calcareous soils. Future studies can focus on the effects of acidifiers on crop yield and the quality of harvested products. Also, in this study, the land was kept fallow, and the effect of acidifiers on GHGs emissions when land is under cropping is still unknown.

## Supplementary Information

Supplementary Table S1.

## Data Availability

The datasets used or analyzed during the current study are available from the corresponding author on reasonable request.
