# Supplementary material for: Effects of acidifiers on soil greenhouse gas emissions in calcareous soils in a semi-arid area
Source: Sci Rep. 2023 Mar 29;13:5113. doi: 10.1038/s41598-023-32127-0 (PMC10060232; doi:10.1038/s41598-023-32127-0)
Supplement: Supplementary file 1 — Supplementary Table S1. [file 41598_2023_32127_MOESM1_ESM.docx]

Table S1. Soil physicochemical properties.

| Soil property | Value | Extractant | Method of measurement | Reference |
| --- | --- | --- | --- | --- |
| Soil texture | Sandy clay loam (Sand:54.11, Silt:19.69, Clay:26.22) | Water | Hydrometer | Gee and Bauder ^1^ |
| Soil organic matter | 0.8% | Oxidation with chromic acid and then titration with ferrous ammonium sulfate | Walkley and Black | Nelson and Summers ^2^ |
| EC | 0.34 dS m^-1^ | 1:2.5 soil: water ratio | pH meter |  |
| Total nitrogen | 0.048 % | H_2_SO_4_ and NaOH | Kjeldahl | Bremner ^3^ |
| Carbonate calcium | 21.6 % | HCl | Calcimeter | Loeppert and Suarez ^4^ |
| Available P | 10.11 (meq L^-1^) | NaHCO_3_ | Olsen | Watanabe and Olsen ^5^ |
| Soil organic carbon | 0.46 % | H_2_SO and potassium dichromate | Walkley and Black | Walkley and Black ^6^ |
| Ca | 09.26 (meq L^-1^) | NH_4_CH_3_CO_2_ | Flame photometry | Rowell ^7^ |
| K | 0.54 (meq L^-1^) | NH_4_CH_3_CO_2_ | Flame photometry | Rowell ^7^ |
| Mg | 16.2 (meq L^-1^) | NH_4_CH_3_CO | Flame photometry | Rowell ^7^ |
| Fe | 1.45 mg kg^-1^ | DTPA | AAS | Lindsay and Norvell^8^ |
| Mn | 3.06 mg kg^-1^ | DTPA | AAS | Lindsay and Norvell ^8^ |
| Zn | 2.3 mg kg^-1^ | DTPA | AAS | Lindsay and Norvell ^8^ |
| Cu | 0.18 mg kg^-1^ | DTPA | AAS | Lindsay and Norvell ^8^ |

References

1 Gee, G. & Bauder, J. Particle size analysis by hydrometer: a simplified method for routine textural analysis and a sensitivity test of measurement parameters. *Soil Science Society of America Journal* **43**, 1004-1007 (1979).

2 Nelson, D. W. & Sommers, L. E. Total carbon, organic carbon, and organic matter. *Methods of soil analysis: Part 3 Chemical methods* **5**, 961-1010 (1996).

3 Bremner, J. M. Nitrogen‐total. *Methods of soil analysis: Part 3 Chemical methods* **5**, 1085-1121 (1996).

4 Loeppert, R. H. & Suarez, D. L. Carbonate and gypsum. *Methods of soil analysis. Part* **3**, 437-474 (1996).

5 Watanabe, F. & Olsen, S. Test of an ascorbic acid method for determining phosphorus in water and NaHCO3 extracts from soil. *Soil Science Society of America Journal* **29**, 677-678 (1965).

6 Walkley, A. & Black, I. A. An examination of the Degtjareff method for determining soil organic matter, and a proposed modification of the chromic acid titration method. *Soil science* **37**, 29-38 (1934).

7 Rowell, D. Soil science method and application, longmangrop. *Limitation Score. Computers & Geosciences* **33**, 1316-1326 (1994).

8 Lindsay, W. L. & Norvell, W. Development of a DTPA soil test for zinc, iron, manganese, and copper. *Soil science society of America journal* **42**, 421-428 (1978).
